# Prediction of Hydration Heat for Diverse Cementitious Composites through a Machine Learning-Based Approach

**DOI:** 10.3390/ma17030715

**Published:** 2024-02-02

**Authors:** Liqun Lu, Yingze Li, Yuncheng Wang, Fengjuan Wang, Zeyu Lu, Zhiyong Liu, Jinyang Jiang

**Affiliations:** 1 School of Materials Science and Engineering, Southeast University, Nanjing 211189, China; 230209138@seu.edu.cn (L.L.); 230238681@seu.edu.cn (Y.L.); fjwang1118@163.com (F.W.); 101012819@seu.edu.cn (Z.L.); liuzhiyong0728@163.com (Z.L.); jiangjinyang16@163.com (J.J.); 2 State Key Laboratory of High Performance Civil Engineering Materials, Jiangsu Research Institute of Building Science Co., Ltd., Nanjing 210008, China; 3 Jiangsu Sobute New Materials Co., Ltd., Nanjing 211103, China; 4 Jiangsu Key Laboratory for Construction Materials, Southeast University, Nanjing 211189, China

**Keywords:** cementitious composites, hydration, characterization, machine learning, prediction

## Abstract

Hydration plays a crucial role in cement composites, but the traditional methods for measuring hydration heat face several limitations. In this study, we propose a machine learning-based approach to predict hydration heat at specific time points for three types of cement composites: ordinary Portland cement pastes, fly ash cement pastes, and fly ash–metakaolin cement composites. By adjusting the model architecture and analyzing the datasets, we demonstrate that the optimized artificial neural network model not only performs well during the learning process but also accurately predicts hydration heat for various cement composites from an extra dataset. This approach offers a more efficient way to measure hydration heat for cement composites, reducing the need for labor- and time-intensive sample preparation and testing. Furthermore, it opens up possibilities for applying similar machine learning approaches to predict other properties of cement composites, contributing to efficient cement research and production.

## 1. Introduction

Cement hydration is a chemical reaction that occurs between cement minerals and water, ultimately resulting in the formation of key hydration products crucial for cement hardening. A key characteristic of this process comes in the form of hydration heat, the heat released during this chemical reaction. It is widely accepted that the comprehension and study of this hydration heat is vitally significant to the production, design, and application of cement materials [1,2,3].

The primary relevance of hydration heat in cement investigations chiefly arises from its critical role in ascertaining structural characterization and evolution. Hydration heat is inextricably linked to the hydration kinetics of cement compositions and plays a pivotal role in defining distinct stages of cement hydration—namely, the initial reaction, induction, acceleration, and deceleration periods. This key identifying feature is widely recognized within the domain of cement-based studies [4,5].

Typically, such distinctions are made noticeable through instances like the notable hydration peak evident close to the 10 h mark, which can be specifically associated with the hydration of alite/C3S [6,7]. This phase forms a significant part of the formation of primary hydration products (C-S-H). By drawing upon the hydration mechanism, hydration heat is perceived as an effective pointer indicative of modifications in the crystal morphology and crystallinity within cement minerals. These changes, reciprocally, have a direct impact on the microstructure and overall performance of cement-based materials [8]. Numerous other studies have explored the variance in heat across different stages to demonstrate the ubiquitous mechanism encompassing various parameters, including but not limited to the water–cement (w/c) ratio [9,10], supplementary cementitious materials (SCMs) [11,12,13,14], and nanomaterials [15,16,17], among others [18,19,20,21]. A thorough understanding of the variations in hydration heat can substantially contribute to the refined design and modification of cementitious materials, which can in turn enhance their mechanical durability and prolong their lifespan.

However, accurately characterizing the hydration heat of cement poses a complex and labor-intensive challenge, largely due to a plethora of influencing factors such as the equipment used, environmental conditions, and the specific methods implemented. For instance, the selection and calibration of instruments for the measurement of hydration heat are vitally important, with isothermal calorimeters and heat flow calorimeters being traditionally used [1,22,23]. These devices require dedicated calibration and consistent maintenance to assure accurate and reliable measurements. Any disruptions or inconsistencies in equipment performance might inevitably result in flawed data, subsequently undermining the reliability of the characterization [3,24,25]. Additionally, the influence of external conditions cannot be overlooked in the process of hydration heat measurement. Environmental elements such as ambient temperature, humidity, and air circulation need to be persistently monitored and controlled to minimize their impact on the measurement. In addition, the hydration process of cement occurs at a relatively slow pace, causing the heat release to span over longer periods. Depending on the type of cementitious material and its composition, the duration of testing can range from mere hours to days or even extend to weeks. This factor makes the task of acquiring comprehensive hydration heat data inherently time-consuming.

Recently, machine learning techniques have surfaced as a promising tool for predicting the properties [26,27] and characterizing the microstructures [28,29,30] of cement composites. Machine learning employs computational algorithms to detect patterns in existing data and to develop predictive models [31]. This data-driven approach allows these algorithms to learn from extensive datasets derived from past experiments, aptly capturing the intricate relationships between cement composition and its properties. There have been various attempts to investigate cement hydration by employing these techniques [32,33,34]. For example, estimates of concrete hydration degrees have been provided through automated machine learning-based microstructure analyses [35], and heat flow rate profiles for cementitious binders containing fly ash have been predicted using deep forest models [36]. Nevertheless, there have yet to be any reported cases of direct hydration heat predictions for various cementitious composites within specific timeframes.

This study presents the development and implementation of a machine learning model specifically designed to predict cement hydration heat. The dataset design included three separate types of cementitious composites, each with distinct mix designs to promote its diversity. Measurements of heat release, gathered experimentally over 24.5 h, were splintered into 18 time intervals to collect data points for each composite. Artificial neural networks, a typical machine learning algorithm, were employed with its network architecture and parameters optimized for maximum prediction accuracy. The performance of the finalized model was validated by comparison with an extra experimental dataset to confirm its precision and efficacy. The model excelled in both accuracy and efficiency, outperforming traditional testing methods. With these trained models, researchers can obtain immediate results, eliminating waits for experiment completion, thereby facilitating significant time and resource savings. Furthermore, potential limitations resulting from environmental sensitivities can be minimized.

## 2. Methods

**Figure 1:**
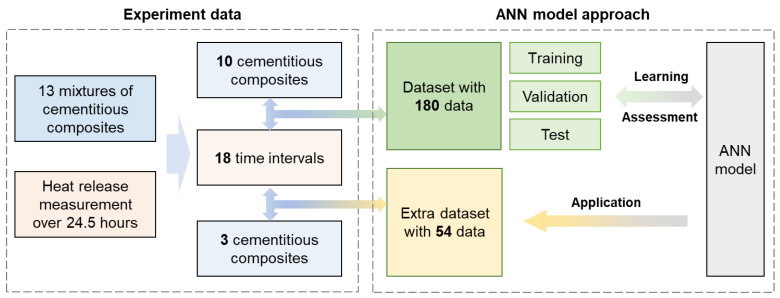
Schematic of the proposed machine learning-based approach for the prediction of hydration heat for diverse cementitious composites.

Figure 1 outlines the workflow used in this study. The main objective was to accurately predict the hydration heat for various types of cementitious composites at specific time intervals. The term “ground truth data” refers to data acquired from experimental measurements of hydration heat. This study leveraged two types of datasets: the standard dataset, which was used for model training and evaluation, and an extra dataset, which was utilized to demonstrate the broader applicability of the predictive model. Each of these stages is further detailed in the following Sections.

### 2.1. Design of Cementitious Composites

To bolster the robustness and broad applicability of the model, 13 distinct cementitious composites were designed, each with varying weight proportions of its components. The cumulative weight of the binders, which includes cement, fly ash, and metakaolin, was kept constant at 1. As denoted in Table 1, samples 1–5 comprised varying water-to-binder (w/b) ratios of ordinary Portland cement pastes. Samples 6–9 were made up of fly ash cement pastes, featuring a consistent w/b ratio but varying amounts of fly ash. Lastly, samples 10–13 encompassed a mixture of Portland cement, fly ash, and metakaolin, all of which consisted of the same w/b ratios and fly ash quantities while differing in the proportions of metakaolin included.

### 2.2. Hydration Heat Data Collection

Heat release from the hydration of cement composites was evaluated using a TAM-Air isothermal calorimeter (TA Instruments, Newcastle, DE, USA). The experiment was performed at a constant temperature of 20 °C, equivalent to room temperature, to maintain consistency given the study’s primary objective of predicting hydration heat at specific time intervals for different cement composites. Measurements of heat release were recorded at 30 s intervals over a duration of 24.5 h. The overall heat release was determined by calculating the area under the curve that plots heat release against time. For subsequent analysis, data points at 18 specific time intervals—1500, 3000, 4500, 6000, 12,000, 18,000, 24,000, 30,000, 36,000, 42,000, 48,000, 54,000, 60,000, 66,000, 72,000, 78,000, 84,000, and 87,000 s—were collected and added to a database.

### 2.3. Data Preprocessing

As shown in Figure 1, the size of the dataset in this study equals the product of the number of composites (13) and the time intervals of hydration heat (18), resulting in 234 total data points. Three composites, samples no. 3, no. 7, and no. 12, were selectively chosen to create an extra dataset of 54 data points to demonstrate the predictive capabilities of the trained model in estimating cement hydration heat. The remaining 10 composites made up a separate dataset with 180 data points for fine-tuning the machine learning model. This dataset was randomly divided into training, validation, and test sets in a specific ratio.

In the traditional machine learning approach, the training set is used for the learning phase, during which parameters such as weights and biases are adjusted to enhance the model’s performance. The validation set is used to identify any potential model overfitting, and based on these results hyperparameters such as the learning rate can be adjusted to avoid overfitting. The test set is used to evaluate the performance of the fully trained model, without any further model training.

In this study, two division scenarios were analyzed: 7:2:1 and 8:1:1. In the 7:2:1 division, the proportion of training, validation, and test data was 70%, 20%, and 10%, respectively. In the 8:1:1 division, 80% of the data were allocated for training, with 10% used for validation and the remaining 10% used for testing.

### 2.4. ANN Model Design and Adjustment

**Figure 2:**
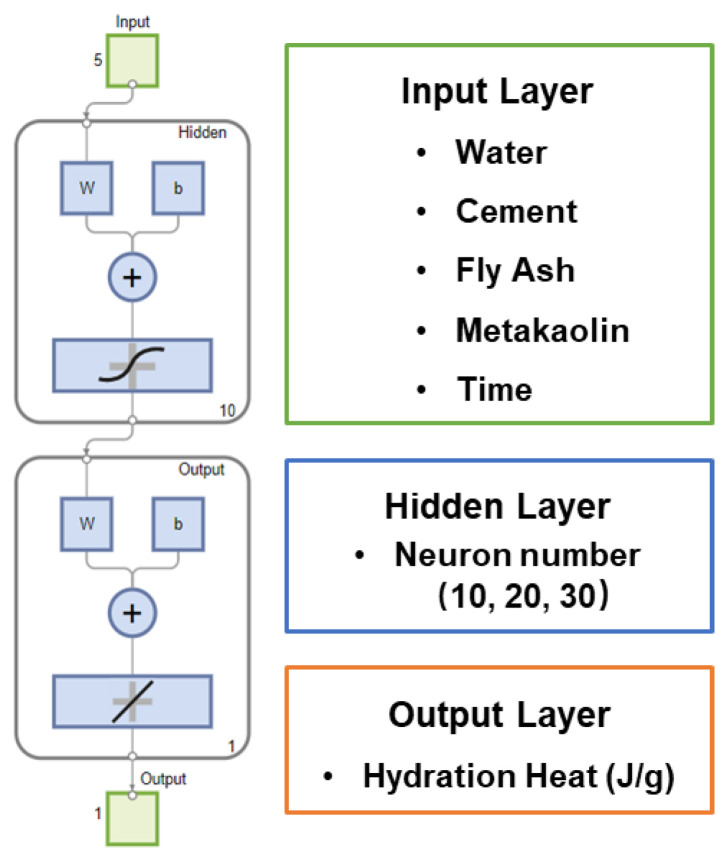
Schematic architecture of the ANN model and parameters.

Figure 2 shows the schematic architecture of the artificial neural network (ANN). The network included five input parameters: water, cement, fly ash, metakaolin, and time. The network’s output was the hydration heat for particular composites at specified times. Preliminary investigations indicated that additional hidden layers could lead to a decline in model performance. Therefore, this study employed a single hidden layer, testing three different neuron counts (10, 20, and 30) to optimize performance. These three ANN models are referred to as NN 10, NN 20, and NN 30, respectively, in subsequent Sections. The Levenberg–Marquardt algorithm was used, paired with a sigmoid activation function.

To prevent overfitting, the epoch was capped at 100 for all scenarios, and early stopping was implemented to halt training if the validation error ceased to decrease. An epoch denotes a full run-through of the entire training dataset in a single cycle when the machine learning model is trained. During an epoch, every training sample in the dataset is processed, and the model’s parameters, such as weight and bias, are updated based on the calculated error. This is a self-learning process that allows the ANN model to adjust its parameters for optimized performance. Also, a 5-fold cross-validation was utilized, and other hyperparameters, like the learning rate and regularization, were adjusted as needed to enhance performance across all scenarios.

In this study, the performance of the model was assessed based on two criteria. One of them is the mean square error (MSE), which is defined by Equation (1), where $Y_{i}$ represents the actual output value and $\hat{Y_{i}}$ represents the predicted value. MSE, or the root mean square error (RMSE), is a widely employed metric for evaluating the performance of machine learning models.

(1) $$MSE=\frac{1}{n}\sum_{i=1}^{n}{\left(Y_{i}-\hat{Y_{i}}\right)}^{2}$$

The other criterion used involves the following steps:

(1) Plot a scatter diagram to compare the predicted output values $\hat{Y_{i}}$ (represented by *Y*) against the true output values $Y_{i}$ (represented by *T*);
(2) Perform a linear fitting for the scatter plot points;
(3) Generate a regression (Equation (2)) relating the predicted output values $\hat{Y_{i}}$ (*Y*) and the true output values $\;Y_{i}$ (*T*).

(2) Y=k×T+b

The regression equation was further evaluated by calculating the coefficient of determination, denoted as R^2^, using Equation (3). In this equation, $\bar{Y}$ represents the average value of the true output.

(3) $$R^{2}=1-\frac{\frac{1}{n}\sum_{i=1}^{n}{\left(Y_{i}-\hat{Y_{i}}\right)}^{2}}{\frac{1}{n}\sum_{i=1}^{n}{\left(Y_{i}-\bar{Y}\right)}^{2}}$$

(4) $$R=\sqrt{1-\frac{\frac{1}{n}\sum_{i=1}^{n}{\left(Y_{i}-\hat{Y_{i}}\right)}^{2}}{\frac{1}{n}\sum_{i=1}^{n}{\left(Y_{i}-\bar{Y}\right)}^{2}}}$$

The performance of the model was evaluated based on the values of k, b, and R. For k and b, perfect prediction is indicated by their values being 1 and 0, respectively. These values represent an exact linear relationship between the predicted and true output values. R is a coefficient of determination ranging between 0 and 1. A higher value of R indicates that the scatter plot points are better aligned with the fitting line and show less fluctuation around it. This suggests a stronger correlation and better fit between the predicted and true output values.

## 3. Results and Discussion

### 3.1. Machine Learning Process

**Figure 3:**
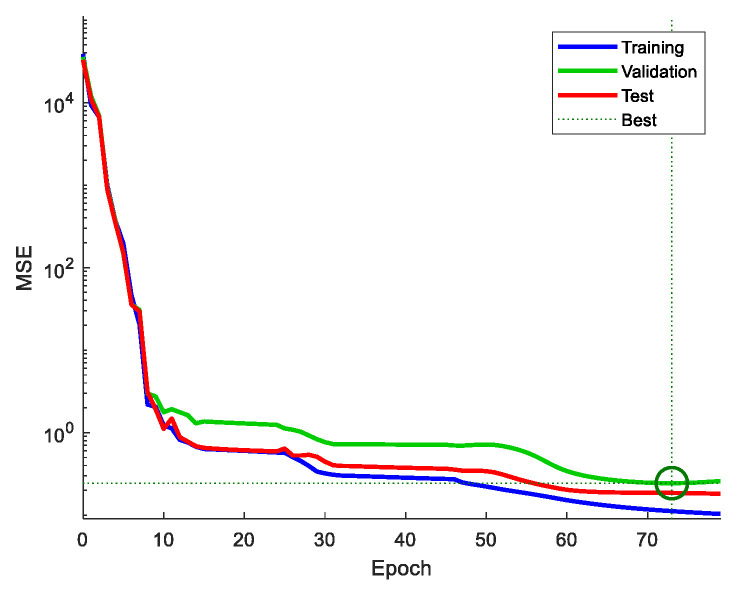
Example of learning termination and model selection.

Figure 3 presents an example of the loss function for the training, validation, and test sets during the learning process. The graph demonstrates that the error drops rapidly until approximately epoch 10, after which it begins decreasing at a slower rate. As the training progresses, the trajectories for the three groups diverge. At around epoch 70, the training error continues to diminish, while the validation error stabilizes, suggesting that further learning could lead to overfitting. Hence, the termination point denoted by the green circle was chosen as the model selection criterion. This approach of stopping the model at this termination point was consistently implemented during the learning process in this study.

### 3.2. ANN Model Performance Analysis

In commonly reported literature, it is expected that the performance of the training and validation sets would be similar. If the validation performance is significantly worse than that of the training set, it indicates overfitting of the model during the learning process. It is generally acceptable for the performance of the test set to be slightly worse than that of the training set. The performance of the entire dataset serves as an indication of how well the ANN model can predict hydration heat for the studied cement composites.

**Figure 4:**
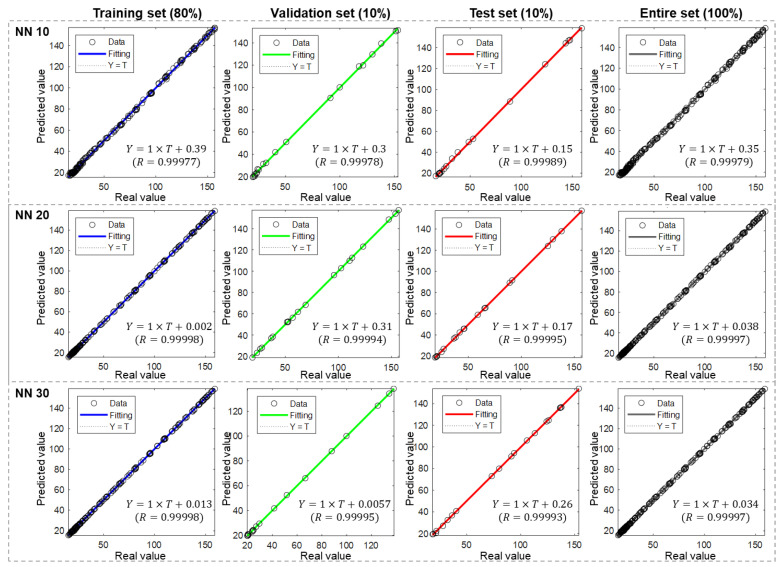
Performance of ANN models with different hidden neuron numbers under an 8:1:1 division ratio.

Figure 4 depicts the performance of ANN models with different numbers of hidden neurons (NNs)—specifically, 10, 20, and 30. Each model was evaluated using four different sets: training, validation, test, and the entire dataset. The division ratio was 8:1:1, which represents that the training, validation, and test set comprised 80%, 10%, and 10% of the entire dataset, respectively. As introduced in Section 2.4, the X axis and Y axis for the scattered data points are real heat value and predicted heat value, respectively.

Through observation, it was noted that all three models exhibited excellent performance across all sets. The scattered points in the scatter plots align closely with the fitting line, as evidenced by the high coefficient of determination (R) values exceeding 0.999. Furthermore, in all cases, the coefficient k is equal to 1, and b is less than 1. These findings suggest that the ANN model is adept at accurately predicting hydration heat at different specific times.

It is important to consider the results in the four sets as they can provide insights into the learning process of the model. For the NN 10 case, the coefficient b in the validation set (0.3) is similar to that in the training set (0.39), and even smaller in the test sets (0.15). This indicates that the trained model captures similarities across the entire dataset. However, the relatively high coefficient suggests that the model has room for improvement.

In contrast, for the NN 20 case, the coefficient b in the training set significantly decreases to 0.002, while it remains relatively unchanged in the other two sets. As a result, the values in the validation and test sets becomes noticeably larger than that in the training set. This suggests the potential occurrence of overfitting during the learning process. In the NN 30 case, the coefficient b has a very small value in the training and validation sets, but it increases to 0.26 in the test set. This situation highlights the importance of dataset division. The model is expected to learn specific features from the training set and may lose accuracy when applied to data outside the set it learned from.

It is worth noting that, in most of the reported literature, performance is evaluated by calculating the R of points with the X = Y line. However, in this study, the performance would not be easily distinguished because almost all points lie on the X = Y line. Therefore, we applied another method as introduced in the Methods Section. Based on the coefficients of regression equation k, b, and R, the slight difference could also be revealed.

**Figure 5:**
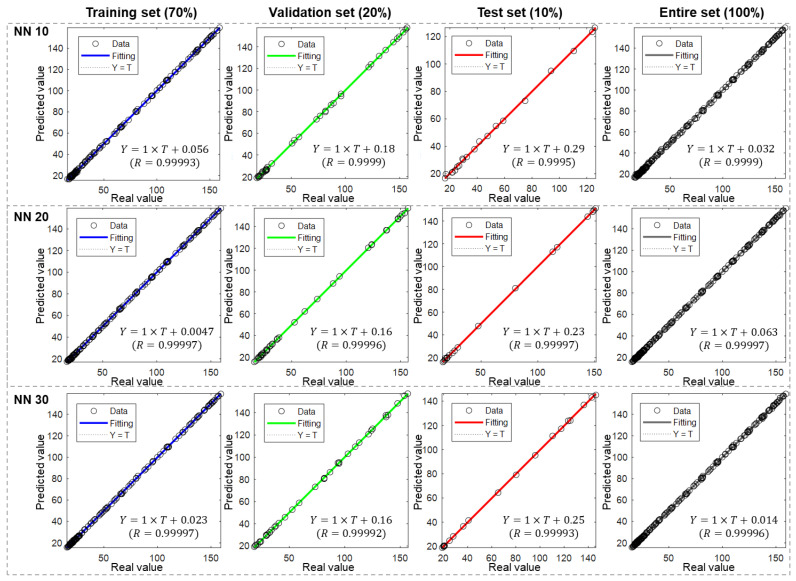
Performance of ANN models with different hidden neuron numbers under a 7:2:1 division ratio.

Figure 5 presents the performance of ANN models with a division ratio of 7:2:1. Similarly, each model was evaluated using four different sets while the proportion of training, validation, and test sets was 70%, 20%, and 10%, respectively.

The overall performance was satisfactory with changes in the proportion of the training and validation sets. In the cases of NN 10 and NN 20, the performance in the validation set improves as the coefficient b decreases from 0.3 to 0.18 and from 0.31 to 0.16, respectively. Meanwhile, the coefficient b for the training set is only 0.056 and 0.0047, respectively. This indicates that the model benefits from having more validation data, leading to improved learning. However, in the NN 30 case, the adjustment in the ratio has a different effect. With a higher validation ratio, both the coefficient b in the training set and the validation set increase. This suggests that the model is not effectively trained based on the current dataset. Overall, the changes in the division ratio highlight the importance of dataset selection and the impact it can have on the model’s performance.

Based on the observations, there was a suspicion that with a hidden neuron number of 10 or 20, the model is capable of learning effectively when the training dataset constitutes 70% or more of the total data. Additionally, having more validation data appears to aid in model learning and helps prevent overfitting. However, as the neuron number increases to 30, the model becomes more complex and demands a larger amount of training data to achieve optimal performance. This suggests that the increased complexity and representation power of the model may require a greater amount of data for training and generalization.

**Figure 6:**
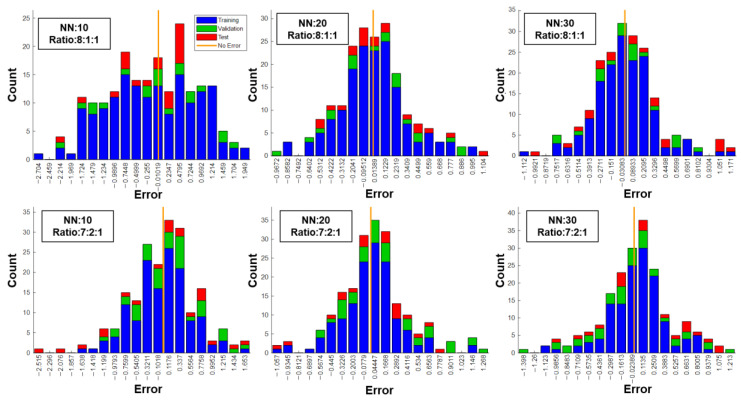
MSE error distribution for all division sets across six cases.

Figure 6 provides the MSE error distribution for all division sets across six cases, offering further assessment of the model’s performance. The X axis is the error value while the Y axis is the count number of samples. Evaluation can be based on the error boundary values and the distribution of samples among 20 bins.

For instance, in the NN 10 case with an 8:1:1 ratio, the error boundary is −2.704 and 1.949. Although the sample distribution is relatively even with more than 10 samples in 13 bins, indicating a lack of strong bias, the model could be considered as not well trained due to this distribution.

In comparison, the NN 10 case with a 7:2:1 ratio has a similar error boundary (−2.515 and 1.653), but the sample distribution is closer to a normal distribution. Notably, the samples located on the boundary mainly belong to the test set, indicating that the model is relatively well trained based on the training and validation sets. However, its performance on other data may be weaker.

Among all cases, the NN 20 case with a 7:2:1 ratio was considered the best model in this study. It has a relatively small error boundary (−1.057 and 1.268), suggesting that the entire dataset has been included and no specific data were excluded during the learning process. Additionally, the distribution can be characterized as a normal distribution with a higher peak and smaller standard deviation.

### 3.3. ANN Model Application

The finalized model was applied to predict an extra dataset, which was completely distinct from the test set. Referring to Table 1, the extra dataset consisted of samples no. 3, no. 7, and no. 12. The exact values of w/c ratio, fly ash ratio, and metakaolin ratio in the extra dataset were not included in the learning dataset. However, it is important to note that the values in the extra dataset fall within the range covered by the learning dataset. Therefore, it can be expected that the constructed ANN model is capable of predicting the hydration heat for the extra dataset as well.

**Figure 7:**
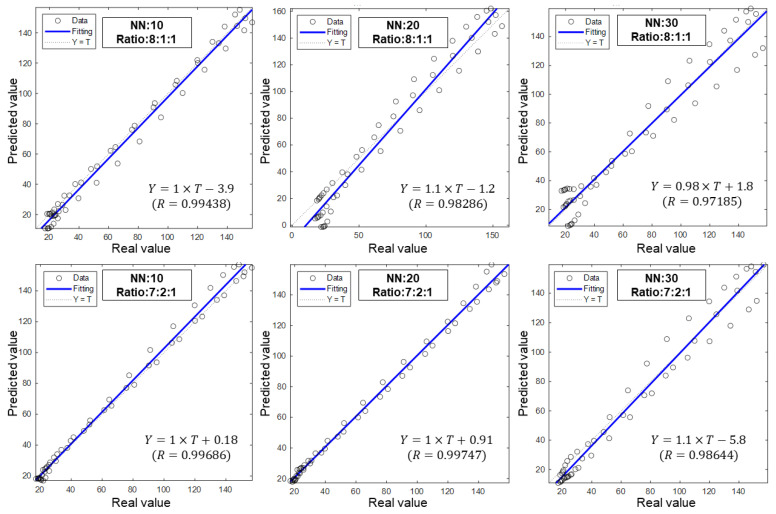
Performance of different ANN models cases on predicting the extra dataset.

Figure 7 displays a scatter diagram and fitting equation of the prediction results for all cases. Observations show that, with a division ratio of 8:1:1, models with more hidden neurons perform worse. For NN 10, the coefficients k and b are 1 and −3.9, respectively, and the R-value is 0.99438. This indicates that the predicted points follow the same trend as the true values, but the exact values are underestimated.

As for NN 20 and NN 30, the coefficient k is 1.1 and 0.98, respectively, and the coefficient b is −1.2 and 1.8, respectively. In these cases, the predicted points no longer exhibit the same trend as the true values, and the fitting line shifts. The corresponding R-squared values are 0.98286 and 0.97185, indicating that the points deviate significantly from the fitting line. It is evident that these models fail to accurately predict the extra dataset. In the case of NN 30, the scatter points form three distinct lines, suggesting that the trained model is only applicable to the learning dataset and lacks generalizability to other data. A similar phenomenon is observed when the division ratio is 7:2:1, although the performance improves slightly with an R-value of 0.98644.

In comparison, NN 10 and NN 20 show more promising results in terms of their predictive performance. Both cases exhibit a coefficient (k) value of 1, while NN 10 has a coefficient (b) of 0.18 and NN 20 has a coefficient (b) of 0.91. These coefficients indicate a desirable linear relationship between the predicted and true output values. Notably, both cases possess coefficients greater than 0.995, suggesting an acceptable variance for the correlation. As a result, the trained model aptly predicts the extra dataset, highlighting the robustness and efficacy of the approach.

Table 2 presents a comparison of MSE results for all datasets examined in this study. The MSE values for the learning datasets (training, validation, and test) are relatively low when compared to the MSE value for the extra dataset, which is expected. These findings align with the detailed analysis provided in the previous sections. Considering the MSE metric, the optimal model for predicting the extra application is the one with 20 hidden neurons, trained using a dataset division ratio of 7:2:1.

It is important to recognize that while the MSE value can provide an overall assessment of a model’s performance during the learning process, it may not directly indicate its suitability for the extra application. For instance, in the case of NN 20 with a ratio of 8:1:1, the MSE values for the learning datasets are lower compared to those of NN 20 with a ratio of 7:2:1. However, the MSE value for extra prediction in the former case is significantly larger than the latter case.

**Figure 8:**
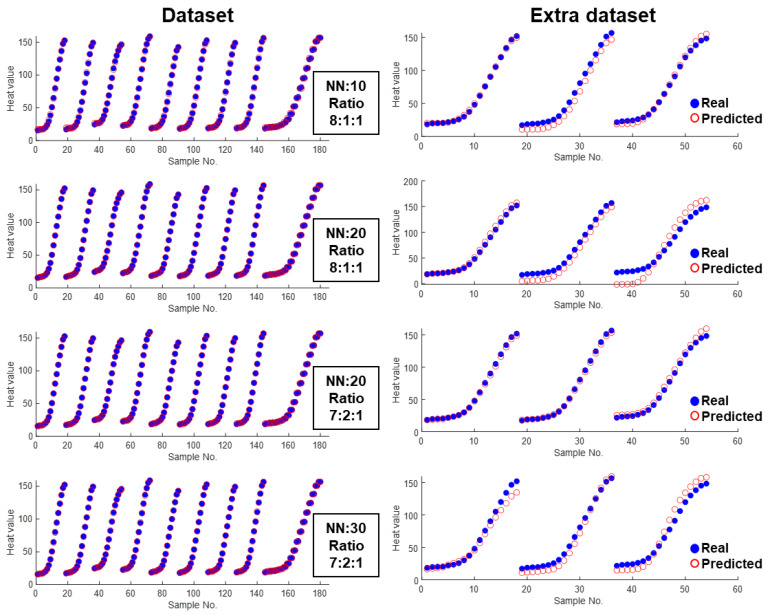
Direct comparison between predicted value and real value for different datasets.

Figure 8 shows a more direct way to compare the model’s performance by plotting the predicted hydration heat value against the real value for each cementitious composite. The best and the worst models under two division ratios are presented. As for the performance on the learning dataset including training, validation, and test, almost all data are well predicted while no obvious differences can be distinguished among those models. However, the performance on the extra dataset is apparently different. In comparison to the conventional MSE assessment, the criteria employed in this study enabled us to identify variations across different learning scenarios, thus providing valuable insights for optimizing the neural network model.

The optimized ANN model exhibits tremendous potential in predicting the hydration heat of diverse cement composites. The results indicate that accurate predictions of hydration heat can be achieved for cement composites falling within the range of the training dataset, for the given composition and time. This breakthrough has the potential to substantially diminish the time and labor required for sample preparation and testing. Furthermore, the proposed machine learning approach holds promise for predicting other properties of various cement composites, extending its applicability beyond hydration heat prediction.

## 4. Conclusions

The characterization of hydration heat during the hydration process of cement composites is crucial in cement material production, design, and usage. However, traditional methods for measuring hydration heat are labor-intensive, time-consuming, and require specialized equipment and controlled environments.

In response to these challenges, this study introduces a machine learning-based approach to predict hydration heat at 18 precise time intervals for 13 different cement composites of varying composition ratios. There are a total of 234 data points, with 180 used for model training and evaluation and 54 used as an additional dataset to demonstrate the model’s robustness. Three diverse model architectures and two dataset division scenarios were compared, and the ANN models’ performances during the learning process were thoroughly analyzed.

The results indicate that the optimized ANN model not only delivers excellent performance on the datasets used during training but also accurately predicts the hydration heat of various cement composites from the extra dataset. This approach has the potential to significantly improve the efficiency of hydration heat measurements for cement composites. Additionally, it underscores the potential that this method may be extended to predict other properties of cement composites.

## Figures and Tables

**Table 1:** Design of cementitious composites in terms of weight ratio.

Sample No.	Water	Cement	Fly Ash	Metakaolin
1	0.32	1	0	0
2	0.34	1	0	0
3	0.36	1	0	0
4	0.38	1	0	0
5	0.4	1	0	0
6	0.38	0.95	0.05	0
7	0.38	0.9	0.1	0
8	0.38	0.85	0.15	0
9	0.38	0.8	0.2	0
10	0.38	0.85	0.1	0.05
11	0.38	0.8	0.1	0.1
12	0.38	0.75	0.1	0.15
13	0.38	0.7	0.1	0.2

**Table 2:** Comparison of MSE results for all datasets.

	Ratio 8:1:1			Ratio 7:2:1		
	NN10	NN20	NN30	NN10	NN20	NN30
**Training**	1.07	0.11	0.10	0.31	0.11	0.13
**Validation**	1.17	0.24	0.18	0.49	0.27	0.36
**Test**	0.62	0.19	0.38	1.26	0.21	0.39
**Extra**	34.23	153.66	129.68	20.35	12.06	81.33

## Data Availability

Data are contained within the article.
